# Retinal laser services in Bhutan: a 3-year national survey

**DOI:** 10.1186/s12886-020-01675-8

**Published:** 2020-10-08

**Authors:** Bhim B. Rai, Michael G. Morley, Pema Zangmo, Thukten Tshering, Abi N. Khatiwara, Paul S. Bernstein, Ted Maddess

**Affiliations:** 1 John Curtin School of Medical Research, Australian National University, Canberra, ACT 2601 Australia; 2 Department of Ophthalmology, JDW National Referral Hospital, Thimphu, Bhutan; 3 Ophthalmic Consultants of Boston, Harvard Medical School, Boston, MA USA; 4 Moran Eye Centre, University of Utah School of Medicine, Salt Lake City, UT USA

**Keywords:** Laser therapy, Laser services in Bhutan, pan-retinal photocoagulation, Photocoagulation, Retinal laser

## Abstract

**Background:**

We conducted this study to report on the indications and types of retinal laser therapy (RLT) performed in Bhutan, knowing which is critical for proper planning and successful delivery of the services.

**Methods:**

We reviewed the laser registers maintained in the laser rooms and vitreoretinal (VR) operating theatres (including paediatric cases managed under anaesthesia) over three years at the national and the two regional referral hospitals (RRHs). Intraoperative laser treatments (endolaser) were excluded. Patient demography, indications and types of RLT were recorded and quantified. Comparisons of the expected and observed frequencies used Chi-squared tests.

**Results:**

A total of 685 patients, including 8 cases of bilateral retinopathy of prematurity (ROP) received RLT. The majority of patients (411 cases, 60.0%, *p* < 0.0001) were males. The mean age was 54.1 ± 14.1 years, median 56 years. The most common indications for RLT were diabetic retinopathy (DR) and diabetic macular oedema (DMO) (542 cases, 66.0%), followed by retinal vein occlusion (RVO) (91 cases, 13.3%). Pan-retinal photocoagulation was the most common type of RLT performed (337 cases, 49.2%), followed by modified grid laser (207 cases, 30.2%), sectoral laser (41 cases, 6.0%), and prophylactic laser photocoagulation (33 cases, 4.8%).

**Conclusions:**

The majority of patients were within working-age. Common indications for RLT were preventable such as DR, DMO and RVO, indicating need to control systemic diseases such as diabetes, hypertension, and dyslipidaemia. Currently, regular RLT is provided only at the national referral hospital in Thimphu, and periodically in the eastern and central RRHs when the retinal specialist visits. There is need to extend the retinal services to the eastern and central RRHs to improve accessibility and patient coverage in these regions challenged with difficult terrain and poor public transport system.

## Background

The therapeutic benefits of laser were first reported in 1954 [1]. This prompted the invention of xenon-arc laser in 1956 for prophylaxis of retinal detachment (RD), but it was limited by poor focus [2]. The first ophthalmic laser was developed in 1960 using a ruby laser [3] marking a big leap forward in the history of retinal laser therapy (RLT) with its blue and green light emissions [4]. Another milestone was the invention of the 532 nm frequency-doubled neodymium-doped yttrium aluminium garnet (Nd:YAG) laser [5]. The macular laser treatment is not considered a standard treatment of care anymore, and has been replaced by intravitreal anti-vascular endothelial growth factor (anti-VEGF) treatment in many places. However, it may probably still be considered as a treatment option if anti-VEGF treatment is not available or limited for socio-economic reasons. It is reported that anti-VEGFs have significantly superior efficacy to laser photocoagulation in diabetic macular oedema (DMO) [6, 7], retinal vein occlusion (RVO) [8], and retinopathy of prematurity (ROP) [9]. In Bhutan, RLT was still considered for retinal disorders such as DMO, diabetic retinopathy (DR), RVO, retinal breaks, ROP, and retinal vasculitis (RV) for economic reasons. The different types of RLT are focal, grid, modified grid, sectoral, scatter, and pan-retinal photocoagulation (PRP).

The aim of the current study was to report on the number, indications and types of RLT, including prophylactic laser photocoagulation (PLP) performed in the whole of Bhutan. This is a complementary article to other studies published by the same authors: first, the pattern of vitreoretinal (VR) diseases in Bhutan, which describes about the ocular comorbidities, associated systemic diseases, clinical data, diagnostic tests performed, and treatment strategies [10]; second, rural-urban difference in myopia prevalence in Bhutan, which gives additional information on patient demography [11]; and third, the surgical management of VR diseases in Bhutan [12]. Since this is the first study of RLT in Bhutan, the results have impacts for policy making on the management of VR diseases in Bhutan and other developing countries. It also serves as a baseline study for future research.

## Methods

### Study design

This retrospective study was approved by the Research Ethics Board of Health (REBH), Ministry of Health, Royal Government of Bhutan, and adhered to the principles of Declaration of Helsinki. Consent was waived by the REBH because only de-identified data were collected.

### Setting

The study was conducted mainly at the VR Subspeciality Clinic (VRSC), Ophthalmology Department, Jigme Dorji Wangchuck National Referral Hospital (JDWNRH), Thimphu, and includes cases receiving RLT in the Eastern Regional Referral Hospital (ERRH) in Monggar, and Central Regional Referral Hospital (CRRH) in Gelephu. The JDWNRH is the apex national referral hospital with tertiary eye-care services in the pyramidal health infrastructure system, supported by the ERRH and CRRH, District Hospitals and Basic Health Units (BHUs) in more remote regions. JDWNRH serves as the main centre providing VR services and as a clinical centre for postgraduate medical students, interns, and optometric and paramedic students.

### Participants

All the patients who received RLT and PLP for VR diseases in Bhutan: JDWNRH, ERRH and CRRH in the study period (01 May 2013 until 30 April 2016) were included. We have reported by the number of patients, not the laser sessions. For example, the PRP per case was usually covered in two sessions, but we counted as one case only. Intraoperative laser treatments such as endolaser during retinal detachment repair or during diabetic vitrectomy were excluded.

### Clinical examination and data collection

The details of medical history taking, clinical examinations, equipment and diagnostic procedures are given in a previous publication [10]. The laser registers maintained in the laser room (LR) in the VR operating theatre (OT) of JDWNRH, ERRH and CRRH were reviewed. Demographic information such as age and gender, number, indications for RLT, and types of RLT were collected.

In paediatric cases, the diagnoses were confirmed by examination under anaesthesia (EUA), following a proper pre-anaesthetic check-up (PAC). If indicated, the RLT was delivered in the same sitting.

### Laser specifications: machine, mode of delivery and types of laser therapies

The majority of patients received RLT in the LR of the out-patient department using a Nd:YAG (VISULAS 532 s, Zeiss Medical Technology, U.K.) frequency doubled laser system. The conventional single-spot slit-lamp delivery method was applied, except in extreme peripheral lesions, where the binocular indirect ophthalmoscopic (BIOM) method was used. For paediatric cases, the green diode laser system (OcuLight Green, IRIDEX, Germany) was used with the BIOM technique. PRP was performed for: generalised retinal involvement such as diabetic retinopathy (DR) and central retinal vein occlusion (CRVO); sectoral laser in branch retinal vein occlusion (BRVO), and hemiretinal BRVO (HRBRVO); and scatter laser in RV. PLP was performed for retinal breaks such as retinal tear and atrophic retinal hole, retinal thinning, chorio-retinal (CR) colobomata, and localised tractional band or tractional retinal detachment (TRD). Confluent laser burns of 2–3 rows around the lesions were performed. Focal laser was delivered for focal pathology such as focal DMO, or lysis of posterior hyaloid membrane in subhyaloid macular haemorrhage. Modified grid or grid focal laser was performed in diffuse DMO. In ROP, the avascular zone including a small rim of normal retina was covered to prevent future TRD.

### Statistical analysis

The data were analysed using MATLAB (2016b, The MathWorks, Natick, MA). Comparisons of the expected and observed frequency of gender used Chi-squared test.

## Results

### Demography

A total of 685 patients received RLT over the study period. The mean age was 54.1 ± 14.1 years (range 0.2 to 87 years), median 56 years. The majority of patients (411 cases, 60.0%, *p* < 0.0001) were males. The infants who received RLT for ROP ranged from 0.2 to 0.4 years. Other demographic details are shown in Table 1.

**Table 1:** Demographic characteristics of patients

Category of patients	Number of patients	Presentation age (mean ± SD)	Minimum age	Median age	Maximum age
All patients, years	685	54.1 ± 14.1	0.2	56	87
Patients treated in LR^a^, years	677	54.8 ± 12.9	10	56	87
Patients treated in OT^b^, years	8	0.28 ± 0.09	0.2	0.25	0.4

^a^*LR* Laser Room, ^b^*OT* Operating Theatre under general anaesthesia

### Indications of retinal laser therapy

DR and DMO were the most common indications for RLT, accounting for 542 cases (66.0% of total laser cases) as shown in Table 2. The second most common indication was for RVO in 91 cases (13.3%). Within the sub-group of RVO, BRVO was the most common diagnosis in 49 cases (53.8% of RVO cases), followed by CRVO in 32 cases (35.2%), and HRBRVO in 10 cases (11.0%). There were 47 cases (6.9%) who received PLP. Retinal breaks in the form of retinal tear due to trauma or traction, and atrophic retinal hole secondary to myopic degeneration were encountered in 36 cases (5.3%) who received RLT. RV was also a common indication seen in 17 cases (2.5%). Eight cases of ROP received RLT bilaterally under general anaesthesia in the VR OT. There were 6 cases of CR coloboma and 4 cases of Coats’ disease. There were 24 miscellaneous cases over the study period. Table 2**.**

**Table 2:** Indications of Laser therapies

Indications	Number	Percentage
Diabetic retinopathy / macular oedema	452	66.0
Retinal Vein Occlusion	91	13.3
Localised TRD^a^ / FV^b^	47	6.9
Retinal breaks (Tear / hole)	36	5.3
Retinal Vasculitis	17	2.5
Retinopathy of prematurity	8	1.2
Chorio-retinal coloboma	6	0.9
Coats’ disease	4	0.6
Others	24	3.5
Total	**685**	**100.0**

^a^*TRD* Tractional Retinal Detachment, ^b^*FVT* Fibro-Vascular Traction

### Types of retinal laser therapy

The different types of RLT performed were PRP, sectoral involving one quadrant of the retina, scatter, PLP, focal, grid, modified grid (grid + focal), and peripheral laser for ROP (Table 3**)**. PRP was the most common type of RLT performed in 337 cases (49.2%) of severe, very severe and proliferative DR, and CRVO. Modified grid laser (combined grid and focal) was the second most common RLT accounting for 207 cases (30.2%) of diffuse DMO associated with focal leakage secondary to microaneurysms. The sectoral laser performed in cases of BRVO and some cases of extensive RV was the third most common type in 41 cases (6.0%). PLP was performed in 33 cases (4.8%); the common indications were retinal breaks, localised tractional bands or localised TRD, and CR colobomata. Twenty-three cases (3.4%) of DMO with no obvious identified leakage site received grid laser. Focal laser was performed in 16 cases (2.3%) of DMO with localised leakage. There were 8 cases of ROP who received RLT bilaterally following PAC and EUA in the VR OT.

**Table 3:** Types of retinal laser therapy

Types	Number	Percentage
Pan-retinal photocoagulation	337	49.2
Modified Grid (grid + focal)	207	30.2
Sectoral	41	6.0
Prophylactic / barrage	33	4.8
Grid	23	3.4
Scatter	20	2.9
Focal	16	2.3
Laser for ROP cases	8	1.2
Total	**685**	**100.00**

## Discussion

Globally, the mortality from non-communicable diseases represented 72.3% of total deaths in 2016 [13]. In Bhutan, the World Health Organization (WHO) reported that 28% of total mortality in Bhutan was due to cardiovascular disease, and another 17% due to other non-communicable diseases in 2016 [14]. The risk factors included harmful use of alcohol, high salt intake, unhealthy diet, physical inactivity, and tobacco use [15]. The prevalence of diabetes and hypertension was 8.2 and 26.0% respectively among the urban Bhutanese men and women, and 54.1% of the diabetes population had hypertension [16]. In our study on pattern of VR diseases in Bhutan the coexistent diabetes and hypertension was the commonest systemic disease associated, followed by isolated hypertension and diabetes [10]. The global prevalence of diabetes is reported at 9.3%, with higher prevalence in the high-income (10.4%) than low-income (4.0%) countries [17].

Although the RLT was an established first line therapy for a number of vascular disorders such as DR and DMO [18], RVO [19], RV [20], ROP [21], and Coats’ disease [22], now it is replaced by anti-VEGF injection in developed countries. In Bhutan it is still performed to treat above mentioned diseases for economic reasons.

Our study of the pattern and presentation of VR diseases in Bhutan [10] and a similar study in Nepal [23] reported that the majority of patients were males (60.3 and 53.0% respectively). That trend was followed here for patients receiving RLT, 60.0% of whom were males.

DR/DMO as the most common VR disorder requiring RLT was expected because it was the third most common VR disease in Bhutan [10]. PRP for DR, and focal, grid, and modified grid for DMO were commonly performed in the developing countries for economic reasons. It is still effective in improving vision and arresting progression of DR [18]. RVO was the second most common indication for RLT because RVO, and its risk factors such as hypertension and diabetes, are common in Bhutan [10]. In DR/DMO and RVO works by reducing the oxygen demand of the outer retina [24].

We successfully performed PLP in cases of localised TRD and rhegmatogenous RD. This is supported by a study reporting on the efficacy of PLP in such cases [25]. Another effective use of laser is for retinal breaks, lattice degeneration and retinal thinning, preventing occurrence of vision-threatening secondary RD [26]. Similarly, we applied PLP for myopic degeneration and retinal tears due to trauma or tractional force. Comparatively, there were very less cases of retinal break availing PLP. This could partly be explained by the low prevalence of myopia in Bhutan [11]. With increasing prevalence of myopia, including pathological myopia, in South-East Asia [27], and high prevalence of myopia, especially among urbanites in Bhutan [11], there is a growing need for screening and treating such complications to prevent vision threatening complications such as RD.

RV was a common indication for RLT in Bhutan [10]. It is popularly known as Eales’ disease and is associated with *Mycobacterium tuberculosis* infection as its etio-pathogenesis [20]. Tuberculosis is still reported to be common in Bhutan, with a total incidence of 149 cases per 100,000 population in 2018 [28]. Some of the patients receiving RLT for RV were Indian nationals working as labourers in Bhutan, and tuberculosis is reported to be common in India [29].

During early development, the retina lacks blood vessels and depends on the primary vitreous for oxygen and nutrients [30]. The vascularisation of the retina commences at 12 weeks and is nearly completed by 36–40 weeks gestation [21]. This process is dependent on hypoxia-induced production of VEGF [31]. Upon premature delivery, the developing retina faces abrupt withdrawal of maternal IGF-1, and faces a relatively hyperoxic external environment. This down regulates VEGF and erythropoietin, resulting in cessation of vascularisation and vaso-obliteration, leaving the peripheral retina avascular. However, increased metabolic activity of the maturing retina becomes hypoxic, upregulating the whole process and leading to retinal neovascularisation and ROP development [21, 32]. The Early Treatment for ROP (ETROP) has reported favourable outcomes with early treatment and has provided new treatment guidelines [33]. The BEAT-ROP study reported treatment with anti-VEGF is superior to the laser treatment because the anterior segment development is present when treated with anti-VEGF and prevents development of very high myopia [9]. We treated the ROP with RLT due to economic constraints.

CR coloboma is a risk for developing RD and higher recurrence after surgery [34]. We performed PLP successfully, which is reported effective to reduce RD [34].

Coats’ disease, characterised by abnormal telangiectasia, retinal exudation, and serous RD, is a vision-threatening disorder that has poor visual prognosis [35]. We managed them with RLT, although other treatment modalities have been advised including anti-VEGF [36].

PRP was the most common RLT performed in our study because VR diseases requiring PRP such as DR and CRVO were very common in Bhutan [10], and in Nepal too [23]. Similarly, modified grid laser was the second most common laser procedure performed because DR and DMO were common [10]. RLT is still the most common and standard treatment for DR and DMO in developing countries for economic reasons [37]. Sectoral RLT was also commonly performed because of higher prevalence of BRVO, HRBRVO, and RV involving extensive retina [10].

The study has some limitations. This is a retrospective and the first exploratory study on retinal laser services in Bhutan. We do not have the data on visual acuities and outcomes of the RLT procedures. Regular RLT was performed only at the JDWNRH, with only periodic visits to the ERRH and CRRH by the retinal specialist for delivering the services. This might have left out some patients in the eastern and central regions despite a good patient referral system.

## Conclusions

Despite being a small country with a population of less than a million [38], Bhutan faces a wide spectrum of VR disorders [10]. Systemic diseases such as diabetes and hypertension which are risk factors for VR disorders are common and increasing [10]. Although the surgical management of VR diseases in Bhutan utilizes diversified treatment approaches [12], the range of RLT performed in this study through the national VR service is still in the early stages of development. Currently, the VR services are mainly provided at the JDWNRH in Thimphu, with periodic visits by the VR team to the ERRH and CRRH. There is need to expand the VR service to the ERRH and CRRH to cover the eastern and central regions which are particularly challenging because of difficult terrain and poor transport services.

## Data Availability

The data have not been placed in any online data storage. The datasets generated and analysed during the study are available upon request from the first author.

## Abbreviations

BCVA: Best corrected visual acuity
BRVO: Branch retinal vein occlusion
CRVO: Central retinal vein occlusion
DMO: Diabetic macular oedema
DR: Diabetic retinopathy
EUA: Examination under anaesthesia
HTR: Hypertensive retinopathy
IOL: Intra-ocular lens
OCT: Optical coherence tomography
PPV: Pars plana vitrectomy
PAC: Pre-anaesthetic checkup
RD: Retinal detachment
ROP: Retinopathy of prematurity
RNFL: Retinal nerve fibre layer
RV: Retinal vasculitis
RVO: Retinal vein occlusion
VEGF: Vascular endothelial growth factor

## References

[1] Meyer-Schwickerath G (1954). Light coagulation; a method for treatment and prevention of the retinal detachment. Albrecht von Graefes Archiv Ophthalmol.

[2] Meyer-Schwickerath G (1956). Prophylactic treatment of retinal detachment by light-coagulation. Trans Ophthalmol Soc U K.

[3] Kapany NS, Peppers NA, Zweng HC, Flocks M (1963). Retinal photocoagulation by lasers. Nature.

[4] L'Esperance FA (1968). An opthalmic argon laser photocoagulation system: design, construction, and laboratory investigations. Trans Am Ophthalmol Soc.

[5] Jalkh AE, Pflibsen K, Pomerantzeff O, Trempe CL, Schepens CL (1988). A new solid-state, frequency-doubled neodymium-YAG photocoagulation system. Arch Ophthalmol.

[6] Regnier S, Malcolm W, Allen F, Wright J, Bezlyak V. Efficacy of anti-VEGF and laser photocoagulation in the treatment of visual impairment due to diabetic macular edema: a systematic review and network meta-analysis. Plos One. 2014;9(7):e102309. 10.1371/journal.pone.0102309 PMC4100770 25029255

[7] Muston D, Korobelnik JF, Reason T, Hawkins N, Chatzitheofilou I, Ryan F (2018). An efficacy comparison of anti-vascular growth factor agents and laser photocoagulation in diabetic macular edema: a network meta-analysis incorporating individual patient-level data. BMC Ophthalmol.

[8] Tan MH, McAllister IL, Gillies ME, Verma N, Banerjee G, Smithies LA (2014). Randomized controlled trial of intravitreal ranibizumab versus standard grid laser for macular edema following branch retinal vein occlusion. Am J Ophthalmol.

[9] Geloneck MM, Chuang AZ, Clark WL, Hunt MG, Norman AA, Packwood EA (2014). Refractive outcomes following bevacizumab monotherapy compared with conventional laser treatment: a randomized clinical trial. JAMA Ophthalmol.

[10] Rai BB, Morley MG, Bernstein PS, Maddess T (2020). Pattern of vitreo-retinal diseases at the national referral hospital in Bhutan: a retrospective, hospital-based study. BMC Ophthalmol.

[11] Rai BB, Ashby RS, French AN, Maddess T. Rural-urban differences in myopia prevalence among myopes presenting to Bhutanese retinal clinical services: a 3-year national study. Graefes Archive Cln Exp Ophthalmol. 2020;258. 10.1007/s00417-020-04891-6. 10.1007/s00417-020-04891-6 32803328

[12] Rai BB, Morley MG, Zangmo P,Tshering T, Khatiwara AN, Bernstein PS, Maddess T. Surgical management of vitreo-retinal diseases in Bhutan: a 3-year national study. New FrontOphthalmol. 2020;6:1–5. 10.15761/NFO.1000251.

[13] Collaborators GBDCoD (2017). Global, regional, and national age-sex specific mortality for 264 causes of death, 1980-2016: a systematic analysis for the global burden of disease study 2016. Lancet.

[14] WHO. Noncommunicable Diseases/Country Profiles. 2016 [cited 2020 01 September]. Available from: https://www.who.int/nmh/countries/btn_en.pdf?ua=1.

[15] Pelzom D, Isaakidis P, Oo MM, Gurung MS, Yangchen P. Alarming prevalence and clustering of modifiable noncommunicable disease risk factors among adults in Bhutan: a nationwide cross-sectional community survey. BMC Public Health. 2017;17. 10.1186/s12889-017-4989-x PMC5740865 29268747

[16] Giri BR, Sharma KP, Chapagai RN, Palzom D (2013). Diabetes and hypertension in urban bhutanese men and women. Indian J Community Med.

[17] Saeedi P, Petersohn I, Salpea P, Malanda B, Karuranga S, Unwin N (2019). Global and regional diabetes prevalence estimates for 2019 and projections for 2030 and 2045: Results from the International Diabetes Federation Diabetes Atlas, 9(th) edition. Diabetes Res Clin Pract.

[18] Group TDRSR (1987). Indications for photocoagulation treatment of diabetic retinopathy: diabetic retinopathy study report no. 14. Int Ophthalmol Clin.

[19] Fekrat S, Goldberg MF, Finkelstein D (1998). Laser-induced chorioretinal venous anastomosis for nonischemic central or branch retinal vein occlusion. Arch Ophthalmol.

[20] Biswas J, Ravi RK, Naryanasamy A, Kulandai LT, Madhavan HN (2013). Eales' disease - current concepts in diagnosis and management. J Ophthalmic Inflamm Infect.

[21] Houston SK, Wykoff CC, Berrocal AM, Hess DJ, Murray TG (2013). Laser treatment for retinopathy of prematurity. Lasers Med Sci.

[22] Kodama A, Sugioka K, Kusaka S, Matsumoto C, Shimomura Y (2014). Combined treatment for Coats' disease: retinal laser photocoagulation combined with intravitreal bevacizumab injection was effective in two cases. BMC Ophthalmol.

[23] Rai BB, Shresthra MK, Thapa R, Essex RW, Paudyal G, Maddess T (2019). Pattern and presentation of Vitreo-retinal diseases: an analysis of retrospective data at a tertiary eye Care Center in Nepal. Asia-Pac J Ophthalmo.

[24] Stefansson E. The mechanism of retinal photocoagulation – how does the laser work? United Kingdom: European Ophthalmic Review; 2009. p. 76–9. 10.17925/EOR.2009.02.01.76.

[25] Shukla D, Maheshwari R, Kim R (2007). Barrage laser photocoagulation for macula-sparing asymptomatic clinical rhegmatogenous retinal detachments. Eye (Lond).

[26] Mastropasqua L, Carpineto P, Ciancaglini M, Falconio G, Gallenga PE (1999). Treatment of retinal tears and lattice degenerations in fellow eyes in high risk patients suVering retinal detachment: a prospective study. Br J Ophthalmol.

[27] Morgan IG, French AN, Ashby RS, Guo X, Ding X, He M (2018). The epidemics of myopia: Aetiology and prevention. Prog Retin Eye Res.

[28] WHO. Bhutan: Tuberculosis profile 2018. Geneva: WHO; 2018. updated 28.01.2020. Available from: http://www.who.int/tb/data/.

[29] Chakraborty AK (2004). Epidemiology of tuberculosis: current status in India. Indian J Med Res.

[30] Goldberg MF (1997). Persistent fetal vasculature (PFV): an integrated interpretation of signs and symptoms associated with persistent hyperplastic primary vitreous (PHPV). LIV Edward Jackson memorial lecture. Am J Ophthalmol.

[31] Shweiki D, Itin A, Soffer D, Keshet E (1992). Vascular endothelial growth factor induced by hypoxia may mediate hypoxia-initiated angiogenesis. Nature.

[32] Smith LE (2008). Through the eyes of a child: understanding retinopathy through ROP the Friedenwald lecture. Invest Ophthalmol Vis Sci.

[33] Jones JG, MacKinnon B, Good WV, Hardy RJ, Dobson V, Palmer EA (2005). The early treatment for ROP (ETROP) randomized trial: study results and nursing care adaptations. Insight.

[34] Gopal L, Badrinath SS, Sharma T, Parikh SN, Shanmugam MS, Bhende PS (1998). Surgical management of retinal detachments related to coloboma of the choroid. Ophthalmology.

[35] Shields JA, Shields CL, Honavar SG, Demirci H, Cater J (2001). Classification and management of coats disease: the 2000 proctor lecture. Am J Ophthalmol.

[36] Sigler EJ, Randolph JC, Calzada JI, Wilson MW, Haik BG (2014). Current management of coats disease. Surv Ophthalmol.

[37] Kozak I, Oster SF, Cortes MA, Dowell D, Hartmann K, Kim JS (2011). Clinical evaluation and treatment accuracy in diabetic macular edema using navigated laser photocoagulator NAVILAS. Ophthalmology.

[38] NSB. Population and Housing Census of Bhutan 2017 Thimphu, Bhutan: National Statistics Bureau of Bhutan; 2017 [updated 2019 January; cited 2019. Available from: http://www.nsb.gov.bt/publication/files/PHCB2017_national.pdf.

